# U.S. Health Insurers' “Main Job” vs. “Side Job”: Data Report 2011–2023

**DOI:** 10.3389/fpubh.2026.1651982

**Published:** 2026-02-10

**Authors:** Yu Lei

**Affiliations:** Barney School of Business, University of Hartford, West Hartford, CT, United States

**Keywords:** enrollment, expense structure, fully-insured plans, health insurance, self-insured plans, third-party administrators

## Introduction

A U.S. district judge on September 4, 2025 approved a settlement requiring Aetna (part of CVS) to pay 4.6 million and its subcontractor Optum (part of UnitedHealth Group) to pay $200,000 to participants in the self-insured employer-sponsored health plans that it administered (1). The two companies were accused of using “dummy codes” to bundle administrative fees with provider charges, thus increasing the total claims cost for employers and out-of-pocket expenses for their employees.

Several other top insurers were also accused of self-dealing in providing administrative services to self-insured employer-sponsored health plans. Cigna was sued for intentionally underpaying out-of-network providers^1^ and then charging “saving fees” to the plaintiff (2). In separate cases, United Healthcare (3), Elevance (formerly known as Anthem)^2^, and Blue Cross Blue Shield of Massachusetts (4) were all sued for making overpayments to providers, resulting in losses to the plaintiffs. United Healthcare (5) was also accused in a separate lawsuit of cross-plan offsetting, i.e., using funds from their self-insured plans to recoup losses incurred in their fully-insured plans.

The growing number of cases against health insurers drew attention to the potentially misaligned incentives between insurers that serve as third-party administrators (TPA) of self-insured plans and their employer clients. The U.S. Internal Revenue Service requires the presence of risk shifting and risk distribution to be present if a transaction is to be considered insurance for federal income tax purpose^3^. An insurance company's “main job” is thus to offer fully-insured plans for which they bear the underwriting risk of claims cost. Administering self-insured plans is a “side job” that does not involve risk transfer as it is the employer client's responsibility to cover claims.

The different risk natures of the two insurance segments may result in differing incentives. In the risk-bearing fully insured business segment, insurers have strong incentives to manage the underwriting risk as they are legally obligated to pay claims even when they have insufficient premium income. In the risk-free^4^ self-insured business segment, insurers are paid a flat per-member-per-month fee to provide contracted administrative services, including using their employer clients' assets to pay claims^5^. Insurers are thus less likely to be as invested in managing the underwriting risk in self-insured plans, which would go against their employer clients' interest in holding down healthcare spending. The overpayment and cross-plan offsetting lawsuits mentioned above provided some anecdotal evidence of the misalignment problem. One report argues that TPA-insurers may also “skimp on costs” (6) or even hide expenses in medical charges as in the Aetna case.

Self-insured plans and fully-insured plans are also regulated differently, which may be another factor contributing to the differences in their claims and expense experiences. Fully-insured plans are subjected to state insurance regulation and all of the regulations under the Patient Protection and Affordable Care Act (ACA). Self-insured plans are exempt from state insurance regulation and are governed at the federal level by the Employee Retirement Income Security Act (ERISA) (7) and some ACA regulations (8)^6^.

The ACA became law on March 23, 2010 and imposed a plethora of new regulations aimed to improve access to quality care at reasonable cost. One of such regulations is the medical loss ratio (MLR) rule which took effect on January 1, 2011 and is enforced by the Centers for Medicare and Medicaid Services (CMS). The MLR rule mandates that carriers spend at least 80% of their premium dollars on medical claims cost and quality improvement expenses in the individual and small group market (85% in the large group market)^7^. If an insurer's MLR falls below the threshold, they must offer rebates to their policyholders.

The MLR requirements only apply to fully-insured plans, not self-insured plans. Several studies examined fully-insured plans' strategies to become MLR compliant. Some found that insurers overstated claims to achieve the MLR threshold. A study (9) found that higher medical claims were the primary reason that insurers in the individual and small group markets whose MLRs were far below the 80% threshold reported larger MLR increases from 2010 to 2011. An analysis of insurer data from 2005 to 2013 (10) reported that individual market insurers increased claims cost by seven percent on average to meet the MLR requirements. Another study (11) estimated that approximately 14 percent of insurers engaged in strategic over-estimates to avoid paying rebates. The rebate amount avoided due to overstated claims was approximately $190 million to $325 million for 2011–2013 (12).

Besides overstating claims, insurers were also found to meet the MLR rule by way of managing expenses. One study (13) reported that fully-insured plans in the individual market reduced their median administrative cost ratio and operating margin between 2010 and 2011 by more than two percentage points each to arrive at a seven-percentage-point increase in their median medical loss ratio.

Two studies compared fully-insured and self-insured plans. One report (14) found that insurers spent the most quality improvement expenses per member in their government plans and the least on their self-insured members, with commercial insurance spending in between these two extremes. Another study (15) had similar findings that self-insured plans incurred lower per-member quality improvement expenses than fully-insured commercial group plans during the study period 2010–2018. The same study also reported that fully-insured plans increased quality improvement expenses to reach the MLR threshold but it was unclear whether the increase was new investment or reallocation of funds from self-insured plans to fully-insured plans.

Given the differing risk natures and applicable regulations in fully-insured plans and self-insured plans, it is important to understand the workings of the self-insured segment in terms of its claims and expense experiences and whether insurers use self-insured plans to cross-subsidize their fully-insured segment (possibly at the expense of their employer clients and plan beneficiaries). To address these questions, we'd need complete claims and expense data from self-insured plans. Insurers though are not required to report claims data for self-insured plans.

Since we only have enrollment and expense data for self-insured plans, we focus our research on examining two things: 1) whether there is any correlation in size between the fully-insured segment and the self-insured segment. We posit that if insurers intend to cross-subsidize between the two segments, there may be a positive relationship between the segment sizes, given the fact that major TPAs are mostly large health insurers. 2) whether there is any difference in expense structure between the two segments. We posit that self-insured plans may spend less on expenses given the anecdotal evidence that some may bundle certain expenses with claims cost and/or skimp on costs to increase net profit.

The rest of the article is organized as follows: we give a brief overview of the prevalence of self-insured plans in the next section, followed by presentation of the data source and analysis results. We conclude the article with discussions and policy implications.

## Increasing Popularity of Self-insured Plans

A majority of people in the U.S. who had health insurance obtained coverage from their or their family members' employers and the latest data available indicated that 53.8% of the insured population were covered by employment-based insurance (also known as group insurance) in 2024 (16). While many employers were already offering health insurance to their employees voluntarily before the ACA became law, the ACA's employer mandate requires all employers with 50 or more full-time employees to offer group insurance beginning in 2015 or pay a per-worker penalty (17). This mandate further makes group insurance a more important issue for employers.

There are two financing options for employers. They may purchase fully-insured plans from commercial insurers, paying a fixed per-worker premium to transfer the risk to their insurers. They could also self-insure their workers' medical expenses, paying claims from their general assets or a trust fund. As employers are not insurance experts, they often outsource the management of health plans to TPAs, which could be insurers or non-insurers.

Both financing options have advantages and disadvantages. With fully-insured plans, employers do not bear the underwriting risk but lack flexibility in tailoring plans to meet their employees' unique health care needs. Additionally, they have no control over premium levels. A Wall St. Journal article (18) cited the benefits consulting firms Mercer and Willis Towers Watson saying that costs for employer coverage are expected to surge around 6.5% for 2024.

Self-insured plans allow employers to design their health plans and invest their premium funds. However, they bear underwriting risk if their workers incur unexpected high claims. The total cost of self-insured plans (including claims cost, administrative expenses, and third-party administrator fees) could be higher than premiums for fully-insured plans.

While employers may favor fully insured plans to transfer the underwriting risk, more than fifty percent of U.S. employers self-insure their health plans to save money, maintain control over benefit design, or to lessen the burden of regulations.

Table 1, which reports our tabulation of the Kaiser Family Foundation's annual Employer Health Benefits Survey results (19), shows that the enactment of the ACA in 2010 has been followed by a steady increase in the percent of covered workers (with group health insurance) in self-insured plans. The 2020 pandemic further fueled the growth of self-insured plans with people canceling or postponing care. Large firms with 200 or more workers were much more likely to self-insure their health plans than small firms. In 2020, 84% of large firms self-insured their health plans, as opposed to 23% for small firms. The percent of covered workers in self-insured plans decreased slightly post-pandemic (when utilization and claims bounced back to pre-pandemic levels), but still stayed at 63% in 2024 across all firm sizes.

**Table 1:** Percent of covered workers in self-insured group insurance.

**Year**	**All firms**	**Small firms (3–199 workers)**	**Large firms (200 + workers)**
2009	57%	15%	65%
2010	59%	16%	63%
2011	60%	13%	64%
2012	60%	15%	62%
2013	61%	16%	61%
2014	61%	15%	62%
2015	63%	17%	63%
2016	61%	13%	61%
2017	60%	15%	79%
2018	61%	13%	81%
2019	61%	17%	80%
**2020**	**67%**	**23%**	**84%**
2021	64%	21%	82%
2022	65%	20%	82%
2023	65%	18%	83%
2024	63%	20%	79%

Data Source: the Kaiser Family Foundation's Employer Health Benefits Survey.

The increasing popularity of self-insured plans was followed by a strong demand for TPAs. While there are many non-insurer third-party administrators, many employers still outsource their self-insured health plan management to health insurers. One study finds that self-insured plans made up a large portion of major health insurers' business mix in 2020, with Cigna having 76% of covered lives in self-insured plans, Aetna 59%, and United Healthcare 42% (6).

Mark Farrah Associates (MFA), a leading data aggregator and publisher, reported that self-insured enrollment has been increasing steadily over time, reaching 133.3 million in 2025 (20). It also found that in 2021 Elevance Health; Health Care Service Corporation (HCSC); Highmark, and Blue Cross Blue Shield of Michigan (BCBS of MI) all collected over $1 billion in income from fees of self-insured plans.

While the prevalence of self-insured plans has been reported, there is limited research about the self-insured segment, especially in its relation to its fully-insured counterpart. This study fills in the gap in literature and offers new insights into the self-insured segment.

## Data

We use the CMS Medical Loss Ratio Data and System Resources public use files (“MLR data”) from 2011 to 2023^8^ to examine health insurers' “main job” (underwriting insurance policies) and “side job” (administering self-insured plans). The MLR data are based on the Supplemental Health Care Exhibit (SHCE), part of health insurers' statutory filings. The SHCE was developed by the National Association of Insurance Commissioners (NAIC) to monitor health insurers' compliance with the MLR rule. The NAIC data containing the SHCE is proprietary. The MLR data is publicly available and looks slightly different from the SHCE. For instance, fees from self-insured plans are reported in line 6 of part 1 in the 2023 MLR data forms^9^ but in line 12 of part 1 in the 2023 SHCE data (21). Another example is that the SCHE directly reports underwriting gain (in line 12) but the MLR form does not.

The MLR data tracks components used to calculate the MLR and rebates (if any) and contain several parts (see footnote 9). For fully-insured plans, insurers are required to report information on premiums, claims, and expenses by lines of insurance.

Self-insured plans are termed uninsured plans in insurers' statutory filings. The CMS defines “uninsured plans” as those “for which a reporting entity, as an administrator, performs administrative services such as claims processing for an employer that is at risk, and accordingly, the administrator has not issued an insurance policy.” ^10^ Since insurers bear no underwriting risk for administering self-insured plans, premiums received and claims paid on behalf of such plans are not reported as premiums or claims for the insurers. They, however, are required to report to CMS fees earned and expenses incurred from managing self-insured plans, as well as other pertinent information such as the number of lives and member months covered in self-insured plans.

The 2011–2023 MLR data included 893 distinct insurers. We conduct our analysis at the firm-year level. We first examine whether there is any correlation between the sizes of fully-insured and self-insured plans and then analyze the two segments' expense structures. Fully-insured plans include all lines of insurance reported, including comprehensive individual market, comprehensive small group market, commercial large group, mini-med individual market, mini-med small group market, mini-med large group market, expatriate small group market, expatriate large group market, student health plans, government program plans, and other health insurance. For definitions of each line of insurance, see CMS filing instructions^11^.

While the mandatory MLR reports were authoritative, there are a few limitations. We note that there were missing values in the data, but it is comparable to the proprietary NAIC data. For instance, our tally of the data indicates that self-insured enrollment increased from 92.3 million in 2011 to 118 million in 2023. A study using the proprietary NAIC data (22) reported an enrollment increase in the self-insured segment from 96 million in 2010 to 118 million in 2022 (the MLR data showed an enrollment of 120 million that year).

Another limitation was that the MLR reports only began in 2011, when the MLR took effect. Insurers however have been administering uninsured plans long before that. Caution must be exercised when attempting to generalize the results reported below.

## Size Correlation

We create two samples to examine the size correlation. The first sample includes observations with non-negative number of lives in fully-insured plans and self-insured plans. The second sample includes observations with non-negative lives in fully-insured plans and non-negative self-insured fees. We include zero-values because some insurers choose to do fully-insured plans or self-insured plans only while others may do both.

We calculate both the Pearson correlation and the Spearman correlation (23). The former uses actual data values and may be influenced by outliers. The latter uses the ranks of data values and is not skewed by outliers. Table 2 reports both correlation coefficients. The left panel shows the correlation between fully-insured enrollment and self-insured enrollment and the right panel shows the correlation between fully-insured enrollment and self-insured fees.

**Table 2:** Correlation between fully insured plans (all lines combined) and self-insured plans.

**Year**	**Fully-insured enrollment vs. Self-insured enrollment**		**Fully-insured enrollment vs. Self-insured fees**	
	**Pearson**	**Spearman**	**Pearson**	**Spearman**
2011	0.1041	0.2486^**^	0.2205^*^	0.3248^***^
2012	0.5782^***^	0.2472^***^	0.6233^***^	0.2800^***^
2013	0.4001^***^	0.3032^***^	0.4577^***^	0.3999^***^
2014	0.4608^***^	0.2520^***^	0.5002^***^	0.2869^***^
2015	0.5694^***^	0.2944^***^	0.6514^***^	0.3554^***^
2016	0.5702^***^	0.4469^***^	0.6534^***^	0.5035^***^
2017	0.4421^***^	0.2967^**^	0.6557^***^	0.2939^***^
2018	0.4555^***^	0.3412^**^	0.6641^***^	0.3596^***^
2019	0.5460^***^	0.2278^**^	0.5755^***^	0.3414^***^
2020	0.5360^***^	0.4301^***^	0.5766^***^	0.5112^***^
2021	0.5341^***^	0.2056^*^	0.5792^***^	0.3294^***^
2022	0.5460^***^	0.2339^**^	0.5966^***^	0.3734^***^
2023	0.5199^***^	0.1541	0.5672^***^	0.2912^***^

Data Source: Author's analysis of the CMS MLR data.

^***^, ^**^, ^*^: significant at the 1%, 5%, and 10% level.

Fully-insured enrollment and self-insured enrollment are shown to be positively correlated and statistically significant in all years (except in 2011 for Pearson correlation and 2023 for Spearman correlation). Both the Pearson and Spearman correlation coefficients between fully-insured enrollment and self-insured fees are positive and statistically significant in all years. Since self-insured fees are usually based on a flat per-member fee, a higher member count will lead to higher total fees if the per-member fee remains constant.

The trend we observe suggests that insurers likely use fully-insured plans and self-insured plans as complements rather than substitutes. One reason could be that insurers may use the same provider network for both segments and higher enrollment in both would enhance their bargaining power with providers. Another likely explanation could be that some insurers may cross-subsidize between the two segments, as the cross-plan offsetting practice by UnitedHealth Group. The MLR rule must be met by lines of insurance and by state in the fully-insured segment, so insurers cannot cross-subsidize across states or lines of insurance within the fully-insured segment. Self-insured plans are exempt from the MLR rule, so insurers may have an incentive to cross-subsidize between self-insured and fully-insured segments, which may incentivize insurers to increase enrollment in both when one is already large.

Our results are also consistent with other studies. A study (24) using the proprietary Clarivate Interstudy (a national proprietary census of health insurance market) reported that in 2021, Health Care Service Corporation, Cigna, CVS Health, UnitedHealth Group, and Elevance Health, collectively enrolled 71 percent of the self-funded segment. Another study (22) using the proprietary NAIC data also found that the top three TPA-insurers in 2022, including CVS, Cigna, and Elevance, were among the five largest fully insured parent organizations that year.

## Expense Structure

The MLR data divides total expenses into three major categories: 1) quality improvement expense (which includes activities to prevent hospital readmission, improve patient safety and reduce medical errors, wellness and health promotion activities, and health information technology expenses related to health improvement); 2) claims adjustment expense (which includes cost containment expenses not included in quality improvement expenses and all other claims adjustment expenses); and 3) general administration expense (which includes direct sales salaries and benefits, agents and brokers fees and commissions, other taxes, other general and administrative expenses, and community benefit expenditures).

To compare the expense structure, we create two samples, one for fully-insured plans and another self-insured plans. For each sample, we include observations with non-negative values of quality improvement expense, claims adjustment expense, and general administration expense. We also remove data values that are higher than the 99^th^ percentile in the three expense categories.

### Expense distribution

Table 3 reports the percentage of total expenses spent on quality improvement, claims adjustment, and general administration. The left panel presents the mean values, and the right panel median values. Both the mean and median values show similar trends.

**Table 3:** Expense Distribution: Self-insured (SI) vs. Fully-insured (FI: All Lines Combined) % of Expenses Spent on Quality Improvement (q), Claims Adjustment (c), and General Administration (g).

**Year**	**Segments**	**Mean values**			**Segments**	**Median values**		
		**%** ***q***	**%** ***c***	**%** ***g***		**%** ***q***	**%** ***c***	**%** ***g***
2011	SI	21%	54%	25%	SI	18%	62%	13%
	FI	8%	17%	75%	FI	7%	16%	76%
	*t*	3.23^***^	7.14^***^	−8.68^***^	χ^2^	4.70^**^	27.24^***^	24.53^***^
2012	SI	21%	61%	18%	SI	16%	64%	10%
	FI	10%	18%	72%	FI	9%	18%	73%
	*t*	3.96^***^	11.14^***^	−14.49^***^	χ^2^	5.87^**^	42.57^***^	37.84^***^
2013	SI	25%	60%	14%	SI	17%	64%	10%
	FI	12%	21%	67%	FI	10%	21%	68%
	*t*	3.41^***^	7.76^***^	−14.74^***^	χ^2^	3.77^*^	25.48^***^	43.58^***^
2014	SI	25%	53%	23%	SI	26%	52%	19%
	FI	11%	18%	71%	FI	9%	19%	74%
	*t*	7.15^***^	13.58^***^	−15.54^***^	χ^2^	37.19^***^	78.20^***^	83.69^***^
2015	SI	25%	52%	23%	SI	27%	50%	19%
	FI	9%	18%	73%	FI	6%	18%	75%
	*t*	9.71^***^	14.26^***^	−17.40^***^	χ^2^	53.85^***^	89.84^***^	84.13^***^
2016	SI	24%	54%	22%	SI	24%	55%	18%
	FI	8%	20%	72%	FI	8%	20%	72%
	*t*	6.25^***^	10.03^***^	−14.24^***^	χ^2^	29.07^***^	44.70^***^	56.98^***^
2017	SI	24%	50%	25%	SI	28%	53%	14%
	FI	12%	21%	67%	FI	10%	21%	69%
	*t*	5.03^***^	8.14^***^	−10.24^***^	χ^2^	16.42^***^	37.53^***^	49.17^***^
2018	SI	22%	52%	27%	SI	21%	55%	15%
	FI	1%	16%	82%	FI	0%	15%	85%
	*t*	8.41^***^	9.37^***^	−12.07^***^	χ^2^	37.15^***^	54.78^***^	54.78^***^
2019	SI	20%	45%	35%	SI	20%	54%	17%
	FI	2%	19%	79%	FI	0%	18%	82%
	*t*	6.95^***^	6.95^***^	−9.16^***^	χ^2^	24.60^***^	39.34^***^	47.00^***^
2020	SI	17%	50%	33%	SI	17%	58%	16%
	FI	4%	19%	77%	FI	0%	17%	77%
	*t*	6.76^***^	7.88^***^	−9.27^***^	χ^2^	21.15^***^	43.99^***^	46.10^***^
2021	SI	16%	47%	37%	SI	17%	57%	18%
	FI	11%	24%	65%	FI	8%	24%	66%
	*t*	2.43^**^	6.13^***^	−5.68^***^	χ^2^	8.64^***^	35.64^***^	33.81^***^
2022	SI	15%	48%	38%	SI	15%	58%	19%
	FI	8%	23%	69%	FI	5%	23%	72%
	*t*	3.98^***^	7.02^***^	−6.95^***^	χ^2^	24.60^***^	35.43^***^	35.43^***^
2023	SI	16%	48%	36%	SI	13%	59%	19%
	FI	8%	22%	70%	FI	4%	23%	73%
	*t*	4.01^***^	7.28^***^	−7.69^***^	χ^2^	26.71^***^	36.97^***^	40.75^***^

Data Source: Author's analysis of the CMS MLR data.

^***^, ^**^, ^*^: significant at the 1%, 5%, and 10% level.

Fully-insured plans spend the highest percentage of expenses on general administration and self-insured plans on claims adjustment. For instance, in 2011, the expense distribution for self-insured plans was an average of 21% on quality improvement, 54% on claims adjustment, and 25% on general administration; for fully-insured plans, it was 8% on quality improvement, 17% on claims adjustment, and 75% on general administration. The median values in the three expense categories for self-insured plans were 18%, 62%, and 13%, respectively. For fully-insured plans, the median percentages were 7% on quality improvement, 16% on claims adjustment and 76% on general administration.

Both segments were also shown to have shifted their expense distribution over the years. For self-insured plans, the average share of quality improvement expenses went from 21% in 2011 to 16% in 2023, peaking at 25% in 2013 and 2014; the average share of claims adjustment expenses reached the highest of 60% in 2013 and dropped from 54% in 2011 to 48% in 2023; the average share of general administration was as low as 14% in 2013 and reached the peak of 38% in 2022 before declining slightly to 36% in 2023. Fully-insured plans also exhibited fluctuations in their expense distribution, with an overall increase in the average share of claims administration and a decline in the other two categories.

Table 3 also shows that self-insured plans on average spend higher percentages of expenses on quality improvement and claims administration but lower percentage on general administration expense than fully-insured plans. The same pattern is also present when we examine the median values. To test whether such differences in each expense category between the two segments are significant, we conduct *t*-test for mean values, and non-parametric Chi-square Mood test (25) for median values. The test statistics are reported in Table 3 and they are significant in every expense category and in all years.

**Figure 1:**
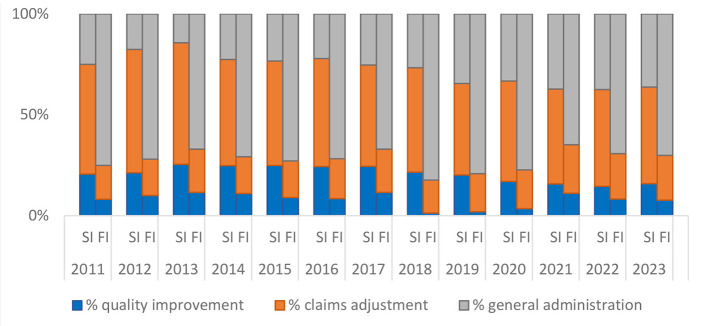
Self-insured (SI) vs. Fully-insured (FI: All Lines Combined): Mean Values of % Expense Distribution. Data Source: Author's analysis of the CMS MLR data.

Figure 1 provides a visual representation of the mean shares. Each bar in the graph is divided into three sections corresponding to the three expense categories. The top section is general administration, the middle claims adjustment, and the bottom quality improvement. The graph indicates that fully-insured plans spend a higher percentage of expenses on general administration but lower percentage on quality improvement and claims adjustment.

### Expense amount

Table 4 reports the mean and median values of total expenses per-member-per-month (PMPM) and PMPM spending on quality improvement, claims adjustment, and general administration. To calculate PMPM values, we divide relevant expense amount by member months. All figures are adjusted for inflation and in 2023 dollars.

**Table 4:** Per-member-per-month (PMPM) Expense Amount (in 2023 Dollars): Self-insured (SI) vs. Fully-insured (FI: All Lines Combined) Amount of Quality Improvement (q), Claims Adjustment (c), General Administration (g), and Overall Expense (o).

**Year**	**Segments**	**Mean Value**				**Segments**	**Median Value**			
		***q***	***c***	***g***	***o***		***q***	***c***	***g***	***o***
2011	SI	1.44	5.02	4.40	10.86	SI	1.18	4.25	0.74	7.03
	FI	1.59	3.67	17.08	22.34	FI	1.50	3.46	15.44	21.17
	*t*	−0.51	1.31	−4.46^***^	−3.41^***^	χ^2^	1.21	0.30	24.44^***^	19.31^***^
2012	SI	2.07	7.83	4.94	14.84	SI	1.73	5.13	0.93	9.29
	FI	3.39	5.95	23.60	32.95	FI	2.64	5.17	22.86	30.88
	*t*	−3.05^***^	1.39	−8.49^***^	−5.32^***^	χ^2^	5.58^**^	0.00	34.87^***^	15.50^***^
2013	SI	1.66	4.99	1.21	7.86	SI	1.34	5.23	0.52	7.54
	FI	3.52	6.80	80.59	90.92	FI	2.68	5.34	20.08	30.56
	*t*	−4.11^***^	−1.76^*^	−2.13^**^	−2.23^***^	χ^2^	8.10^***^	0.17	42.33^***^	37.21^***^
2014	SI	1.63	4.31	1.24	7.18	SI	1.33	3.45	0.87	5.67
	FI	3.77	6.44	25.98	36.19	FI	2.87	4.88	23.38	31.30
	*t*	−5.91^***^	−2.79^***^	−16.29^***^	−13.76^***^	χ^2^	29.18^***^	2.75^*^	95.46^***^	78.04^***^
2015	SI	1.68	4.05	1.73	7.46	SI	1.54	3.24	1.04	6.49
	FI	2.26	5.25	25.01	32.51	FI	1.96	4.85	22.66	28.58
	*t*	−2.66^***^	−2.23^**^	−13.28^***^	−12.81^***^	χ^2^	2.15	9.56^***^	86.08^***^	74.42^***^
2016	SI	1.63	4.10	1.86	7.59	SI	1.43	3.98	1.12	7.52
	FI	2.45	6.34	26.27	35.07	FI	2.24	5.52	21.73	31.88
	*t*	−3.42^***^	−3.83^***^	−11.69^***^	−11.48^***^	χ^2^	9.31^***^	4.14^**^	66.24^***^	66.24^***^
2017	SI	2.09	4.82	3.59	10.51	SI	1.96	4.04	0.85	8.46
	FI	3.75	6.84	24.32	34.92	FI	3.91	7.30	22.76	37.15
	*t*	−5.34^***^	−3.09^***^	−10.62^***^	−10.45^***^	χ^2^	30.05^***^	6.54^**^	58.89^***^	53.42^***^
2018	SI	1.80	4.99	2.30	9.09	SI	1.77	4.38	1.10	8.40
	FI	0.28	6.23	48.17	54.69	FI	0.00	6.26	34.83	41.88
	*t*	5.99^***^	−1.57	−3.51^***^	−3.46^***^	χ^2^	26.21^***^	6.70^***^	50.73^***^	33.20
2019	SI	1.63	5.87	7.72	15.22	SI	1.26	3.97	1.12	8.27
	FI	0.56	7.17	34.06	41.79	FI	0.00	6.50	34.14	41.23
	*t*	3.60^***^	−1.03	−4.19^***^	−3.77^***^	χ^2^	10.88^***^	6.58^***^	50.08^***^	32.75^***^
2020	SI	1.48	5.11	8.10	14.69	SI	1.32	4.01	1.12	7.68
	FI	0.69	6.10	26.92	33.71	FI	0.00	4.83	23.19	27.83
	*t*	2.97^***^	−0.94	−2.96^***^	−2.88^***^	χ^2^	4.03^**^	0.26	58.59^***^	30.84^***^
2021	SI	1.95	6.72	9.05	17.72	SI	1.77	4.29	1.34	8.12
	FI	3.77	8.85	26.16	38.79	FI	3.30	8.62	23.16	37.16
	*t*	−4.07^***^	−1.37	−2.74^***^	−3.21^***^	χ^2^	4.72^**^	10.30^***^	54.14^***^	49.17^***^
2022	SI	2.33	7.61	12.67	22.61	SI	1.49	4.16	1.38	7.81
	FI	3.40	10.60	35.65	49.65	FI	2.88	11.26	31.37	47.69
	*t*	−1.51	−1.71^*^	−2.59^**^	−2.93^***^	χ^2^	21.03^***^	21.03^***^	63.72^***^	53.33^***^
2023	SI	2.10	8.35	3.41	13.86	SI	1.45	4.49	1.52	8.37
	FI	3.38	12.87	46.29	62.54	FI	2.50	11.42	41.69	59.26
	*t*	−1.85^*^	−2.04^**^	−10.46^***^	−9.25^***^	χ^2^	8.15^***^	29.68^***^	81.25^***^	81.25^***^

Data Source: Author's analysis of the CMS MLR data.

^***^, ^**^, ^*^: significant at the 1%, 5%, and 10% level.

In 2011, self-insured plans spent a total of $10.86 in expense per-member-per-month, of which $1.44 on quality improvement, $5.02 on claims adjustment, and $4.40 on general administration. In contrast, fully-insured plans incurred a total expense of $22.34 per-member-per-month, with $1.59 on quality improvement, $3.67 on claims administration, and $17.08 on general administration.

We observe similar trends for both mean and median values. Self-insured plans spend the most dollars on claims adjustment and fully-insured plans on general administration, which is consistent with the expense distribution discussed earlier.

Fully-insured plans incur higher general administration expenses and higher total expenses than self-insured plans in all years (both the *t*-tests of difference in mean values and Mood tests of difference in median values are significant every year).

With regard to quality improvement expenses, fully-insured plans are also shown to spend more than self-insured plans in almost all years, an observation consistent with previous studies (14, 15). The differences between mean values are significant every year except in 2011 and 2022; the differences between median values are significant every year except in 2011 and 2015.

For claims adjustment expenses, fully-insured plans also spend more than self-insured plans but test statistics (*t* and Chi-square) are only significant in a little over half the study period.

**Figure 2:**
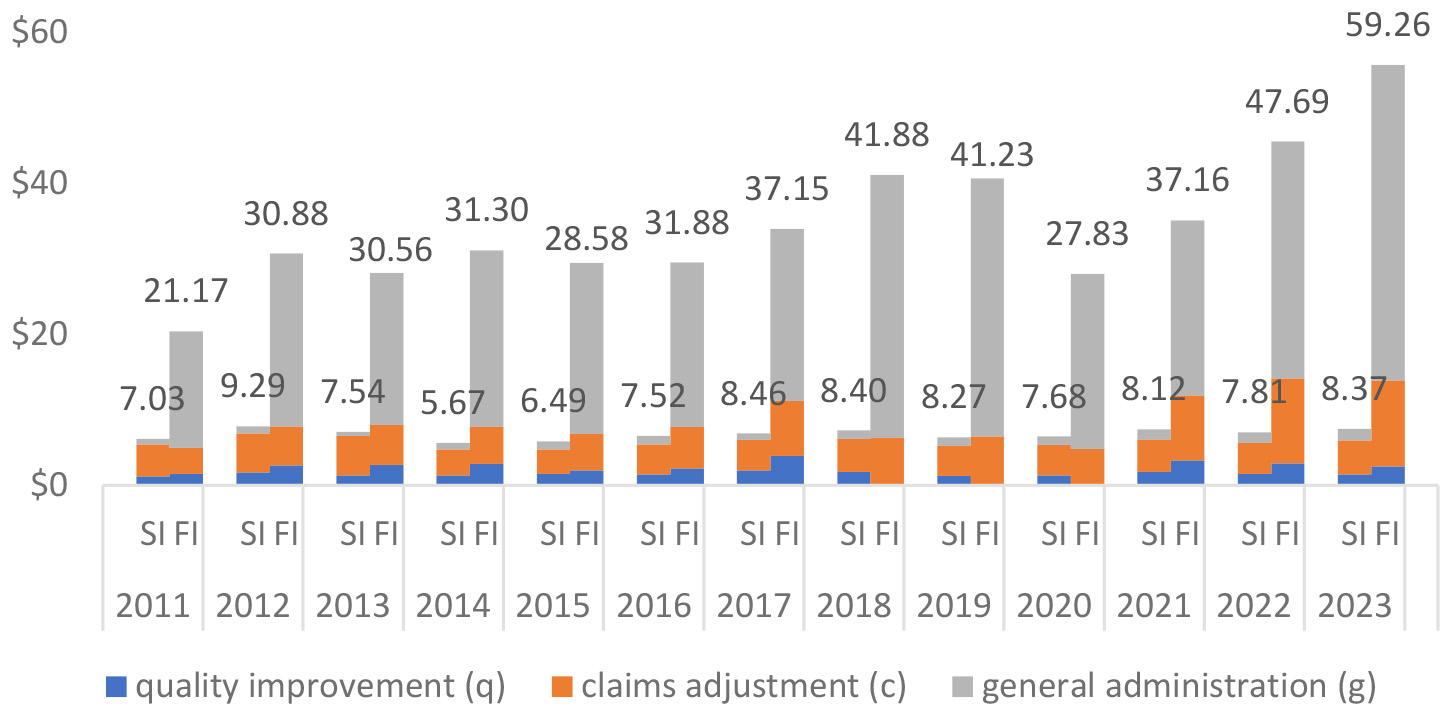
Self-insured (SI) vs. Fully-insured (FI: All Lines Combined) Median Value of PMPM Expense Amount (in 2023 $). Data Source: Author's analysis of the CMS MLR data.

Figure 2 shows the median values. The numerical values shown in the graph are median values of PMPM total expenses, and the bars indicate the breakdown of expenses. Each bar is divided into three sections corresponding to the PMPM dollar amounts spent on quality improvement, claims adjustment, and general administration. Note that the bar heights are not necessarily the same as the figures on top because median values of the three expense categories do not necessarily add up to the median value of the total expenses. The graph shows that fully-insured plans spend the most on general administration and self-insured plans on claims adjustment. We also note that there is not much variation in the median value of PMPM total expenses incurred by self-insured plans as they ranged from $5.67 in 2014 to $9.29 in 2012. Fully-insured plans however exhibit more fluctuation and an overall increasing trend, with the median values of PMPM total expenses increasing from the lowest of $21.17 in 2011 to the highest of $59.26 in 2023.

## Robustness Study

We also replicate our study using a different set of fully insured plans that removes the individual markets. In other words, the new set of fully insured plans covers commercial group (including comprehensive small group market, commercial large group, mini-med small group market, mini-med large group market, expatriate small group market, and expatriate large group market) and the Federal Employees Health Benefits (FEHB) Program, which provides benefits to nearly 8.3 million federal enrollees and dependents and is the largest employer sponsored health plans of its kind (26).

Table 5 presents the size correlation results. Both the Pearson and the Spearman correlation coefficients are positive and significant in all years between fully-insured enrollment and self-insured fees. As for the correlation between fully-insured enrollment and self-insured enrollment, the Pearson correlation coefficients are positive and significant except in 2011 and the Spearman correlation coefficients are also positive and significant except in 2018 and 2019. Again, this shows that a larger fully-insured segment is associated with a larger self-insured segment.

**Table 5:** Correlation between fully insured plans (Group Market Only) and self-insured plans.

**Year**	**Fully-insured Enrollment vs. Self-insured Enrollment**		**Fully-insured Enrollment vs. Self-insured Fees**	
	**Pearson**	**Spearman**	**Pearson**	**Spearman**
2011	0.1070	0.2675^**^	0.2120^*^	0.2800^**^
2012	0.2988^***^	0.3958^***^	0.3158^***^	0.4083^***^
2013	0.2092^**^	0.3056^***^	0.2835^***^	0.3715^***^
2014	0.2676^***^	0.2001^**^	0.2905^***^	0.2361^***^
2015	0.4852^***^	0.2733^***^	0.6011^***^	0.3315^***^
2016	0.4463^***^	0.3558^***^	0.5595^***^	0.4410^***^
2017	0.3952^***^	0.2721^**^	0.5826^***^	0.3131^***^
2018	0.4259^***^	0.1558	0.6000^***^	0.3221^***^
2019	0.4621^***^	0.1456	0.4878^***^	0.3273^***^
2020	0.4945^***^	0.3095^***^	0.5344^***^	0.4133^***^
2021	0.5005^***^	0.1811^*^	0.5449^***^	0.3147^***^
2022	0.5249^***^	0.2129^**^	0.5732^***^	0.4008^***^
2023	0.5394^***^	0.2182^**^	0.5852^***^	0.3938^***^

Data Source: Author's analysis of the CMS MLR data.

^***^, ^**^, ^*^: significant at the 1%, 5%, and 10% level.

**Figure 3:**
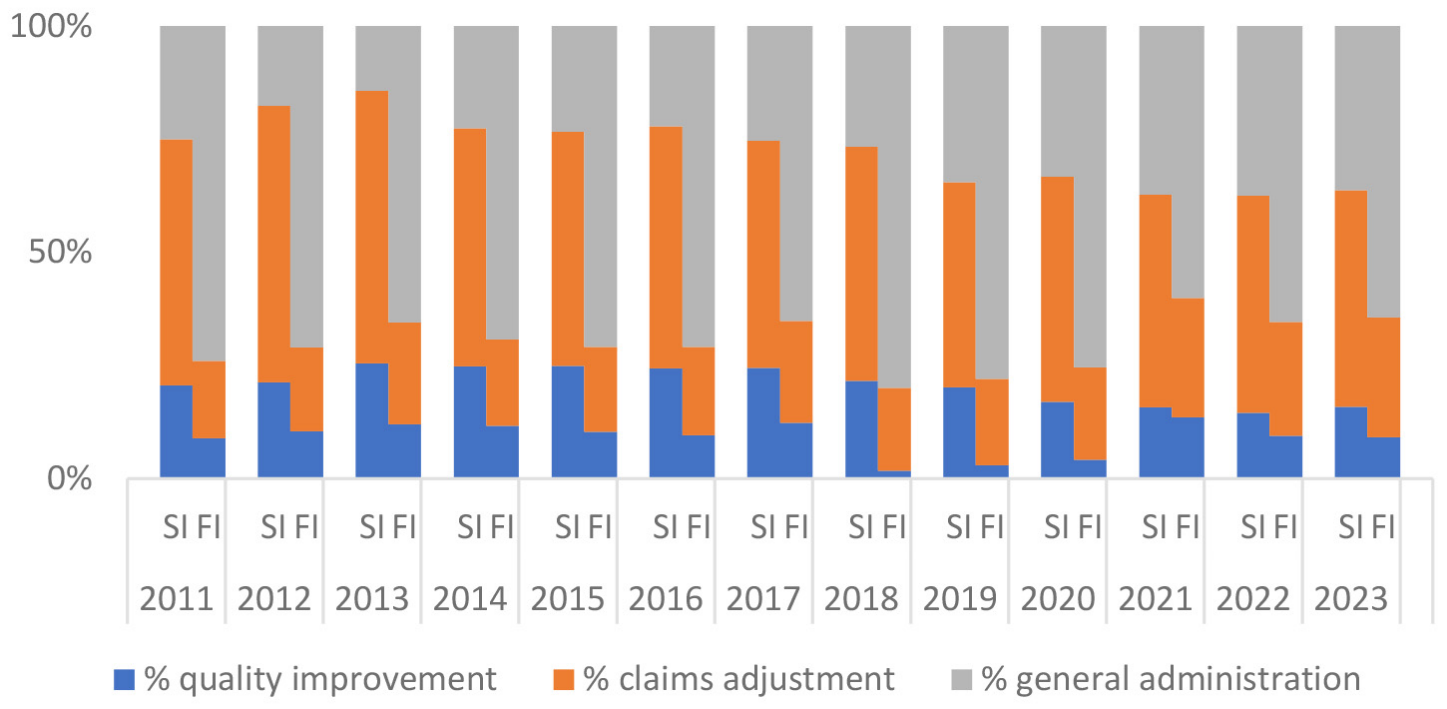
Self-insured (SI) vs. Fully-insured (FI: Group Market Only): Mean Values of % Expense Distribution. Data Source: Author's analysis of the CMS MLR data.

Table 6 shows the same trend of expense distribution as in Table 3 with self-insured plans spending a higher percentage of expenses on quality improvement and claims adjustment but lower percentage on general administration than fully-insured plans. Both the *t*-test and Mood test statistics are significant in all years (except in 2021 with mean value for quality improvement expense share and 2013 with median value for quality improvement expense). Figure 3 presents a visual depiction of the mean values.

**Table 6:** Expense Distribution: Self-insured (SI) vs. Fully-insured (FI: Group Market Only) % of Expenses Spent on Quality Improvement (q), Claims Adjustment (c), and General Administration (g).

**Year**	**Segments**	**Mean values**			**Segments**	**Median values**		
		**%** ***q***	**%** ***c***	**%** ***g***		**%** ***q***	**%** ***c***	**%** ***g***
2011	SI	21%	54%	25%	SI	18%	62%	13%
	FI	9%	17%	74%	FI	8%	17%	75%
	*t*	3.04^***^	7.20^***^	−8.60^***^	χ^2^	4.07^**^	25.42^***^	25.42^***^
2012	SI	21%	61%	18%	SI	16%	64%	10%
	FI	11%	19%	71%	FI	9%	18%	71%
	*t*	3.75^***^	11.02^***^	−14.19^***^	χ^2^	4.23^**^	42.78^***^	38.03^***^
2013	SI	25%	60%	14%	SI	17%	64%	10%
	FI	12%	23%	65%	FI	11%	21%	66%
	*t*	3.30^***^	7.45^***^	−14.05^***^	χ^2^	2.60	26.24^***^	43.24^***^
2014	SI	25%	53%	23%	SI	26%	52%	19%
	FI	12%	19%	69%	FI	9%	20%	73%
	*t*	6.69^***^	11.44^***^	−14.86^***^	χ^2^	38.06^***^	79.54^***^	82.90^***^
2015	SI	25%	52%	23%	SI	27%	50%	19%
	FI	10%	19%	71%	FI	7%	19%	74%
	*t*	8.11^***^	13.92^***^	−15.71^***^	χ^2^	45.61^***^	90.55^***^	84.80^***^
2016	SI	24%	54%	22%	SI	24%	55%	18%
	FI	10%	20%	71%	FI	8%	20%	71%
	*t*	5.66^***^	10.09^***^	−13.63^***^	χ^2^	25.69^***^	49.13^***^	56.98^***^
2017	SI	24%	50%	25%	SI	28%	53%	14%
	FI	12%	22%	65%	FI	10%	22%	69%
	*t*	4.57^***^	7.55^***^	−9.39^***^	χ^2^	15.90^***^	36.82^***^	45.72^***^
2018	SI	22%	52%	27%	SI	21%	55%	15%
	FI	2%	18%	80%	FI	0%	15%	85%
	*t*	8.13^***^	8.53^***^	−11.22^***^	χ^2^	29.26^***^	45.26^***^	53.78^***^
2019	SI	20%	45%	35%	SI	20%	54%	17%
	FI	3%	19%	78%	FI	0%	18%	82%
	*t*	6.33^***^	6.80^***^	−8.77^***^	χ^2^	15.85^***^	38.36^***^	44.03^***^
2020	SI	17%	50%	33%	SI	17%	58%	16%
	FI	4%	20%	75%	FI	0%	17%	77%
	*t*	6.32^***^	7.11^***^	−8.54^***^	χ^2^	16.81^***^	43.03^***^	43.03^***^
2021	SI	16%	47%	37%	SI	17%	57%	18%
	FI	14%	26%	60%	FI	10%	27%	63%
	*t*	1.07	5.48^***^	−4.63^***^	χ^2^	4.82^**^	31.05^***^	27.40^***^
2022	SI	15%	48%	38%	SI	15%	58%	19%
	FI	10%	25%	65%	FI	6%	25%	66%
	*t*	3.07^***^	6.17^***^	−5.98^***^	χ^2^	16.47^***^	28.99^***^	30.92^***^
2023	SI	16%	48%	36%	SI	13%	59%	19%
	FI	9%	27%	64%	FI	5%	27%	66%
	*t*	3.18^***^	6.03^***^	−6.31^***^	χ^2^	18.65^***^	34.35^***^	38.06^***^

Data Source: Author's analysis of the CMS MLR data.

^***^, ^**^, ^*^: significant at the 1%, 5%, and 10% level.

Table 7 reports the PMPM amount spent (in 2023 dollars) in the three expense categories. We note similar trends to those presented in Table 5. Fully-insured plans spend more on general administration and total expenses (the test statistics are significant in all years). Except in 2011 and 2020 (for mean values) and 2020 (for median values), fully-insured plans also incur higher quality improvement expenses than self-insured plans with significant test statistics. As for claims adjustment expenses, fully-insured plans are shown to spend more with statistical significance in all years except in 2011, 2012, and 2019 for mean values and 2011–2013 for median values.

**Table 7:** Per-member-per-month (PMPM) Expense Amount (in 2023 Dollars): Self-insured (SI) vs. Fully-insured (FI: Group Market Only) Amount of Quality Improvement (q), Claims Adjustment (c), General Administration (g), and Overall Expense (o).

**Year**	**Segments**	**Mean Value**				**Segments**	**Median Value**			
		***q***	***c***	***g***	***o***		***q***	***c***	***g***	***o***
2011	SI	1.44	5.02	4.40	10.86	SI	1.18	4.25	0.74	7.03
	FI	1.98	4.26	17.70	23.94	FI	1.86	4.19	15.62	23.04
	*t*	−1.65	0.71	−5.13^***^	−4.13^***^	χ^2^	4.32^**^	0.02	25.42^***^	18.46^***^
2012	SI	2.07	7.83	4.94	14.84	SI	1.73	5.13	0.93	9.29
	FI	3.62	6.69	26.87	37.17	FI	2.76	5.58	24.23	33.12
	*t*	−3.60^***^	0.85	−9.45^***^	−6.29^***^	χ^2^	7.59^***^	0.15	38.03^***^	15.50^***^
2013	SI	1.66	4.99	1.21	7.86	SI	1.34	5.23	0.52	7.54
	FI	3.82	8.01	96.17	108.00	FI	2.95	6.10	20.91	33.64
	*t*	−4.68^***^	−2.79^***^	−2.26^***^	−2.40^***^	χ^2^	13.12^***^	1.37	43.24^***^	36.87^***^
2014	SI	1.63	4.31	1.24	7.18	SI	1.33	3.45	0.87	5.67
	FI	4.00	7.22	27.55	38.77	FI	3.06	6.18	24.62	34.31
	*t*	−6.67^***^	−3.65^***^	−18.43^***^	−15.70^***^	χ^2^	41.05^***^	10.77^***^	82.90^***^	90.86^***^
2015	SI	1.68	4.05	1.73	7.46	SI	1.54	3.24	1.04	6.49
	FI	2.54	5.65	25.98	34.16	FI	2.31	5.22	23.18	31.02
	*t*	−3.86^***^	−2.98^***^	−13.28^***^	−13.49^***^	χ^2^	13.71^***^	13.71^***^	84.80^***^	79.92^***^
2016	SI	1.63	4.10	1.86	7.59	SI	1.43	3.98	1.12	7.52
	FI	2.81	7.04	30.44	40.29	FI	2.74	7.07	24.47	33.51
	*t*	−5.01^***^	−4.99^***^	−12.44^***^	−12.72^***^	χ^2^	19.44^***^	16.56^***^	56.98^***^	66.24^***^
2017	SI	2.09	4.82	3.59	10.51	SI	1.96	4.04	0.85	8.46
	FI	4.01	7.77	25.90	37.68	FI	3.95	8.37	26.57	40.16
	*t*	−6.34^***^	−4.05^***^	−11.29^***^	−11.97^***^	χ^2^	33.54^***^	12.91^***^	45.72^***^	58.11^***^
2018	SI	1.80	4.99	2.30	9.09	SI	1.77	4.38	1.10	8.40
	FI	0.54	7.79	50.46	58.79	FI	0.00	6.58	35.00	42.77
	*t*	3.90^***^	−3.30^***^	−3.62^***^	−3.74^***^	χ^2^	16.95^***^	10.25^***^	53.78^***^	41.01^***^
2019	SI	1.63	5.87	7.72	15.22	SI	1.26	3.97	1.12	8.27
	FI	0.89	7.85	35.54	44.27	FI	0.00	7.15	34.56	43.16
	*t*	1.95^*^	−1.57	−4.44^***^	−4.19^***^	χ^2^	5.19^**^	7.48^***^	44.03^***^	40.71^***^
2020	SI	1.48	5.11	8.10	14.69	SI	1.32	4.01	1.12	7.68
	FI	1.45	7.24	31.74	40.43	FI	0.00	5.96	28.90	36.46
	*t*	0.07	−2.41^**^	−3.70^***^	−3.96^***^	χ^2^	1.41	7.01^***^	43.03^***^	53.82^***^
2021	SI	1.95	6.72	9.05	17.72	SI	1.77	4.29	1.34	8.12
	FI	4.53	10.18	27.02	41.73	FI	4.03	10.90	25.34	39.37
	*t*	−5.51^***^	−2.27^**^	−2.84^***^	−3.67^***^	χ^2^	19.01^***^	22.17^***^	27.40^***^	56.24^***^
2022	SI	2.33	7.61	12.67	22.61	SI	1.49	4.16	1.38	7.81
	FI	3.97	12.68	59.90	76.55	FI	3.01	12.88	31.21	46.61
	*t*	−2.27^**^	−2.86^***^	−1.82^*^	−2.04^**^	χ^2^	20.69^***^	23.92^***^	30.92^***^	69.16^***^
2023	SI	2.10	8.35	3.41	13.86	SI	1.45	4.49	1.52	8.37
	FI	3.99	16.21	55.31	75.52	FI	2.89	17.07	42.20	63.46
	*t*	−2.62^***^	−3.50^***^	−6.91^***^	−7.47^***^	χ^2^	18.29^***^	43.30^***^	38.06^***^	84.86^***^

Data Source: Author's analysis of the CMS MLR data.

^***^, ^**^, ^*^: significant at the 1%, 5%, and 10% level.

**Figure 4:**
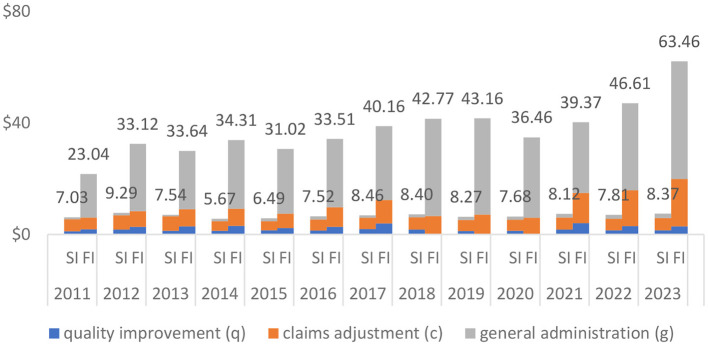
Self-insured (SI) vs. Fully-insured (FI: Group Market Only) Median Value of PMPM Expense Amount (in 2023 $). Data Source: Author's analysis of the CMS MLR data.

Figure 4 shows the median values. When we compare it to Figure 2, we also note that fully insured plans that only include group markets incur higher total expenses and in each expense category in almost all years than fully insured plans that include all lines (both individual and group markets).

## Discussion and Policy Implication

Using a publicly available data source, we examine self-insured plans and fully-insured plans in terms of their enrollment and expense structure. We find that higher enrollment in the fully-insured segment is associated with higher enrollment and fees in the self-insured segment, an observation consistent with the fact that top health insurers in the fully-insured segment are also major TPAs of self-insured plans.

Additionally, we find that self-insured plans spend less expenses per member per month when compared to fully-insured plans. Our study however does not prescribe a reason. It is unclear whether lower expenses in the self-insured segment are insurers skimping on cost, hiding expenses in claims, reallocating funds from self-insured plans to cross-subsidize their fully-insured plans, or being more cost-effective. More data about insurers' TPA practices would be required to decipher the causes for lower expenses in self-insured plans. Future study may link the MLR data to insurers' financial statements to examine what kind of insurers (with regard to specific firm characteristics) may be more likely to lower expenses in their self-insured segment than fully-insured segment. Research is also warranted to study the possible consequences of the lower expenses in self-insured plans for employers and plan beneficiaries.

Our study also shows that insurers exhibit different expense structure in its risk-bearing segment and risk-free segment. Self-insured plans spend a higher percentage on quality improvement and claims adjustment but a lower percentage on general administration than fully-insured plans. The results are largely the same regardless of whether we include individual markets in the fully insured plans. Assuming there is no expense manipulation or cross-subsidization between the fully-insured and self-insured segments, this would be welcome news for employers if their TPAs focus more on quality improvement and claims adjustment than general administration, which is another topic for future study.

While our observational study does not set out to examine the causes and consequences of the reported patterns, our findings shed new lights on the self-insured segment of the health insurance industry, an understudied field despite the segment's prevalence. As more employers self-insure their employers' healthcare cost, it is imperative that they monitor their TPA's management of their health plans. The ERISA requires employer to fulfill their fiduciary duty to plan beneficiaries. Mis-management of health plans will expose the employer to lawsuits by their own employees (27). Wells Fargo (28) and JP Morgan (29) are just two examples of companies being sued by their workers for overspending on health benefits that resulted in higher out of pocket expenses for employees. Both companies used TPA to manage healthcare benefits.

Our study helps employers, policymakers, and researchers understand more about self-insured plans' expense behavior. To address the bigger question raised in the introduction section regarding whether insurers cross-subsidize fully-insured plans at the expense of self-insured plans, we would also require claims data and other pertinent information such as how TPAs compensate their subtractors. The Consolidated Appropriations Act of 2021 (30) removed the gag clause that'd prevent TPAs from sharing claims data, but the law does not obligate TPAs to provide such data (31). TPAs are reportedly still hindering employer efforts to monitor claims cost (32). Pulse of the Purchaser, a national survey of employers, reported that a third of employers surveyed in 2025 are still struggling to get their TPAs to provide information about their own claims data (33). Without such data, employers would not be able to examine the claims cost and expenses, much less evaluate whether their TPAs are acting in their best interest.

Policymakers may consider requiring TPAs to provide claims data and other pertinent information to allow employers to evaluate TPA's effectiveness in managing their health plans. The MLR data used in the study does not contain the proprietary financial information of insurers (such as their assets and liabilities). More publicly available data will also assist researchers in the study of the self-insured segment.

## Data Availability

The raw data supporting the conclusions of this article will be made available by the authors, without undue reservation.

## Footnotes

1
https://www.millerchevalier.com/sites/default/files/resources/General_Alerts/Cigna%20Complaint.pdf

2
https://www.dwt.com/-/media/files/blogs/employment-advisor/2023/08/85w.pdf?rev=3bc489ecd81f49468585fa77fcc778e6&hash=E009DD69CC32251B26FEA5717C6C81B6

3
https://www.irs.gov/pub/lanoa/pmta01392_7369.pdf

4 In the sense of free of underwriting risk.

5
https://mightywellhealth.com/wp-content/uploads/2022/12/Planstin-ASA.pdf

6
https://www.cms.gov/cciio/resources/files/downloads/opt_out_memo.pdf

7
https://www.cms.gov/marketplace/resources/data/medical-loss-ratio-data-systems-resources

8
https://www.cms.gov/marketplace/resources/data/medical-loss-ratio-data-systems-resources

9
https://www.cms.gov/files/document/2023-mlr-reporting-form.xlsx

10
https://www.cms.gov/cciio/resources/files/downloads/mlr-annual-form-instructions051612.pdf

11
https://www.cms.gov/files/document/2024-mlr-form-instructions.pdf

## References

[1] Pifer Parduhn R. Aetna, Optum Settle ‘Dummy Codes' Case for $8.4M (2025). Available online at: https://www.healthcaredive.com/news/aetna-optum-dummy-codes-settlement/759469/ (accessed December 18, 2025).

[2] The ERISA Edit: More Litigation Involving Health Plan Savings Fees. (2023). Available online at: https://www.millerchevalier.com/publication/erisa-edit-more-litigation-involving-health-plan-savings-fees (Accessed December 18, 2025).

[3] Clancy v. United Healthcare Insurance Company Document #173. Available online at: https://www.courtlistener.com/docket/60189081/173/clancy-v-united-healthcare-insurance-company/ (Accessed December 18, 2025).

[4] Haefner M. Lawsuit accuses BCBS of Massachusetts of systematically allowing overpayments (2021). Available online at: https://www.beckershospitalreview.com/legal-regulatory-issues/lawsuit-accuses-bcbs-of-massachusetts-of-systematically-allowing-overpayments/ (Accessed December 18, 2025).

[5] Scott v. UnitedHealth Group, Inc. Document #46. Available online at: https://www.courtlistener.com/docket/17356781/46/scott-v-unitedhealth-group-inc/

[6] Walker L. Inside Big Health Insurers' Side Hustle (2021). Available online at: https://tradeoffs.org/2021/09/23/inside-big-health-insurers-side-hustle/

[7] *Employee Retirement Income Security Act (ERISA)*. Available online at: https://www.dol.gov/general/topic/retirement/erisa (Accessed December 18, 2025).

[8] Market Reforms (ACA & HIPAA) Non-Grandfathered Plan Provisions. Available online at: https://www.cms.gov/cciio/resources/forms-reports-and-other-resources/downloads/market-reforms-aca-and-hipaa-non-grandfathered-plan-provisions.pdf (Accessed December 18, 2025).

[9] Abraham JM Karaca-Mandic P Simon K. How has the affordable care act's medical loss ratio regulation affected insurer behavior? Med Care. (2014) 52:370–7. doi: 10.1097/MLR.0000000000000091 24535023

[10] Cicala S Lieber EMJ Marone V. Regulating markups in US health insurance. Am Econ J Appl Econ. (2019) 11:71–104. doi: 10.1257/app.20180011

[11] Eastman EM Eckles DL Van Buskirk A. Accounting-based regulation: Evidence from health insurers and the Affordable Care Act. Account Rev. (2021) 96:231–59. doi: 10.2308/tar-2019-0173

[12] Plummer E Wempe WF. Do health insurers manage their medical loss ratios? At what cost? J Insur Regul. (2021) 40:1–37. doi: 10.52227/23553.2021

[13] McCue M Hall M Liu X. Impact of medical loss regulation on the financial performance of health insurers. Health Aff. (2013) 32:1546–51. doi: 10.1377/hlthaff.2012.1316 24019358

[14] McCue MJ Hall M. Health insurers' financial performance and quality improvement expenditures in the affordable care act's second year. Med Care Res Rev. (2015) 72:113–22. doi: 10.1177/1077558714563172 25524866

[15] Born PH Sirmans ET Steinorth P. Health insurers' use of quality improvement expenses to achieve a minimum medical loss ratio requirement. J Risk Insur. (2023) 90:123–54. doi: 10.1111/jori.12413

[16] Health insurance coverage in the United States (2024). Available online at: https://www.census.gov/library/publications/2025/demo/p60-288.html (Accessed December 18, 2025).

[17] Economic Report Card for the Affordable Care Act's Employer Mandate (2021). Available online at: https://trumpwhitehouse.archives.gov/articles/economic-report-card-affordable-care-acts-employer-mandate/ (Accessed December 18, 2025).

[18] Wilde Mathews A. Employers' Health-Plan Costs to Swell (2023). Available online at: https://www.wsj.com/public/resources/documents/ROT8DV3i7ppUyhF6koI2-WSJNewsPaper-9-8-2023.pdf

[19] Employer Health Benefits Survey (2024). Available online at: https://www.kff.org/health-costs/report/2024-employer-health-benefits-survey/ (Accessed December 18, 2025).

[20] Second quarter enrollment trends; a segment-by-segment comparison (2025). Available online at: https://www.markfarrah.com/mfa-briefs/second-quarter-enrollment-trends (Accessed December 18, 2025).

[21] NAIC. Supplemental Health Care Exhibit Report (2023). Available online at: https://content.naic.org/sites/default/files/hcs-zb-22.pdf (Accessed December 18, 2025).

[22] Abraham JM Cook AC Sirmans ET. Prevalence and profits of insurers in the administrative services only market serving self-insured employers. Health Aff . (2024) 43:1655–63. doi: 10.1377/hlthaff.2024.00359 39626143

[23] Spearman correlation. Available online at: https://www.sciencedirect.com/topics/mathematics/spearman-correlation (Accessed December 18, 2025).

[24] Meiselbach MK Marr J Wang Y. Enrollment trends in self-funded employer-sponsored insurance. Health Aff . (2024) 43:91–7. doi: 10.1377/hlthaff.2023.00690 38190590

[25] Pan Y Caudill S Li R Caldwell KL. Median and quantile tests under complex survey design using SAS and R. Comput Methods Programs Biomed. (2014) 117:292–97. doi: 10.1016/j.cmpb.2014.07.007 25123100 PMC5708173

[26] Federal Employees Health Benefits (FEHB) Program Carriers. Available online at: https://www.opm.gov/healthcare-insurance/carriers/fehb/ (Accessed December 18, 2025).

[27] Pinder J. Law firms take aim at employers over spending on healthcare plans (2023). Available online at: https://clearhealthcosts.com/blog/2023/12/law-firms-take-aim-at-employers-over-spending-on-healthcare-plans/

[28] Wells Fargo sued over mismanagement of health care plan reported by remy samuels. Available online at: https://www.plansponsor.com/wells-fargo-sued-over-mismanagement-of-health-care-plan/ (Accessed December 18, 2025).

[29] JP Morgan targeted in ERISA healthcare fiduciary breach suit. Available online at: https://www.napa-net.org/news/2025/3/jpmorgan-targeted-in-erisa-healthcare-fiduciary-breach-suit/ (Accessed December 18, 2025).

[30] FAQs about consolidated appropriations act 2021 implementation part 69. Available online at: https://www.dol.gov/agencies/ebsa/about-ebsa/our-activities/resource-center/faqs/aca-part-69 (Accessed December 18, 2025).

[31] Handorf K Monahan CH Watts K. Third-Party administrators—the middlemen of self-funded health insurance. Health Aff Forefront (2025). doi: 10.1377/forefront.20250514.32345

[32] Employers still struggle to access insurers' health cost data sara hansard (2024). Available online at: https://news.bloomberglaw.com/daily-labor-report/employers-still-struggle-to-access-insurers-health-cost-data

[33] Pulse of the purchaser 2025 survey results (2025). Available online at: https://www.nationalalliancehealth.org/resources/pulse-of-the-purchaser-2025-survey-results/ (Accessed December 18, 2025).

